# Case Report: Low cardiac output syndrome with multisystem complications following total repair of tetralogy of fallot

**DOI:** 10.3389/fped.2025.1681837

**Published:** 2026-01-06

**Authors:** Daendy Nova Setia, Tartila, Yogi Prawira

**Affiliations:** 1 Pediatrics Subspecialist Education Program, Child HealthScience, Medical Faculty, University of Indonesia, Cipto Mangunkusumo Hospital, Jakarta, Indonesia; 2 Pediatric Emergency and Critical Care Division, Department of Child Health, Faculty of Medicine, University of Indonesia, Cipto Mangunkusumo Hospital, Jakarta, Indonesia

**Keywords:** tetralogy of fallot, low cardiac output syndrome, junctional ectopic tachycardia, atrial thrombosis, cortical blindness, pediatric cardiac surgery

## Abstract

**Background:**

Tetralogy of Fallot (ToF) repair in infants may be complicated by low cardiac output syndrome (LCOS), which can precipitate rare multisystem complications. Junctional ectopic tachycardia (JET), atrial thrombosis, and cortical blindness are underrecognized sequelae requiring multidisciplinary management.

**Case presentation:**

We report a 23-month-old male with ToF who developed LCOS post-surgery, followed by refractory JET, left atrial thrombus, embolic occipital infarction, and transient cortical blindness. LCOS was defined by elevated lactate, low ScvO₂, hypotension, and oliguria. JET was managed with amiodarone, ivabradine, magnesium, and targeted temperature control. Thrombosis resolved with heparin, yet cortical infarct occurred. Neuroprotective therapy included piracetam and mannitol.

**Outcome:**

Partial visual recovery was observed by POD-30. Follow-up echocardiography showed improved RV function and resolution of arrhythmia. The patient was discharged on POD-35 with improving neurologic status.

**Conclusion:**

LCOS can trigger a cascade of cardiac, thrombotic, and neurologic complications. Early recognition and multidisciplinary intervention are essential. This case highlights the potential reversibility of cortical blindness and the importance of comprehensive postoperative surveillance.

## Introduction

Tetralogy of Fallot (ToF) is among the most prevalent cyanotic congenital heart defects, typically requiring surgical repair in early infancy. Despite substantial improvements in surgical techniques and postoperative care, children undergoing ToF repair remain vulnerable to a host of perioperative complications.

One critical concern is low cardiac output syndrome (LCOS), characterized by inadequate tissue perfusion despite sufficient preload—a phenomenon that can precipitate widespread systemic dysfunction. In pediatric populations, limited cardiopulmonary reserve amplifies LCOS severity, increasing the risk of arrhythmias, thromboembolic events, and neurologic compromise.

This case highlights not only the systemic cascade triggered by LCOS, but also the rare occurrence of cortical blindness with complete recovery—an outcome that reinforces the importance of early neuroprotective intervention and multidisciplinary care.

## Case presentation

A 23-month-old male presented with persistent cyanosis during crying, poor oral intake, and failure to thrive. ToF was diagnosed at 4 months of age. He underwent complete surgical repair comprising ventricular septal defect closure, pulmonary stenosis resection, transannular patch placement, and patent foramen ovale preservation. Cardiopulmonary bypass time was 84 min; aortic cross-clamp time was 40 min. Initial physical examination revealed prolonged capillary refill, cool extremities, and diminished urine output, consistent with low perfusion. Neurologic assessment was limited due to sedation, but pupillary reflexes were intact and no focal deficits were noted prior to POD-17.

**Figure 1:**
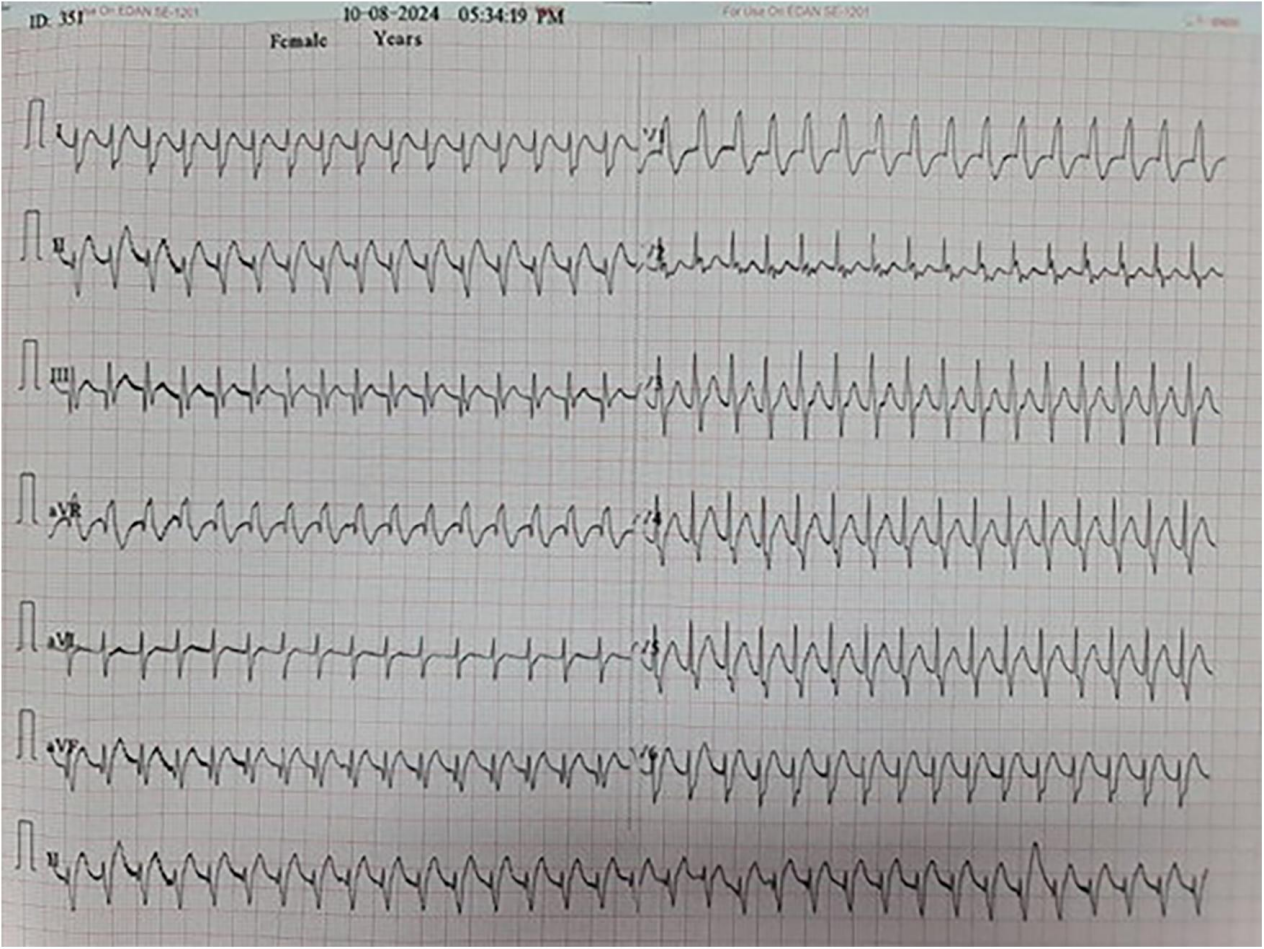
Electrocardiogram showing junctional ectopic tachycardia (JET) with narrow QRS complexes and atrioventricular dissociation. The ventricular rate exceeds the atrial rate, consistent with postoperative JET following Tetralogy of Fallot repair.

**Figure 2:**
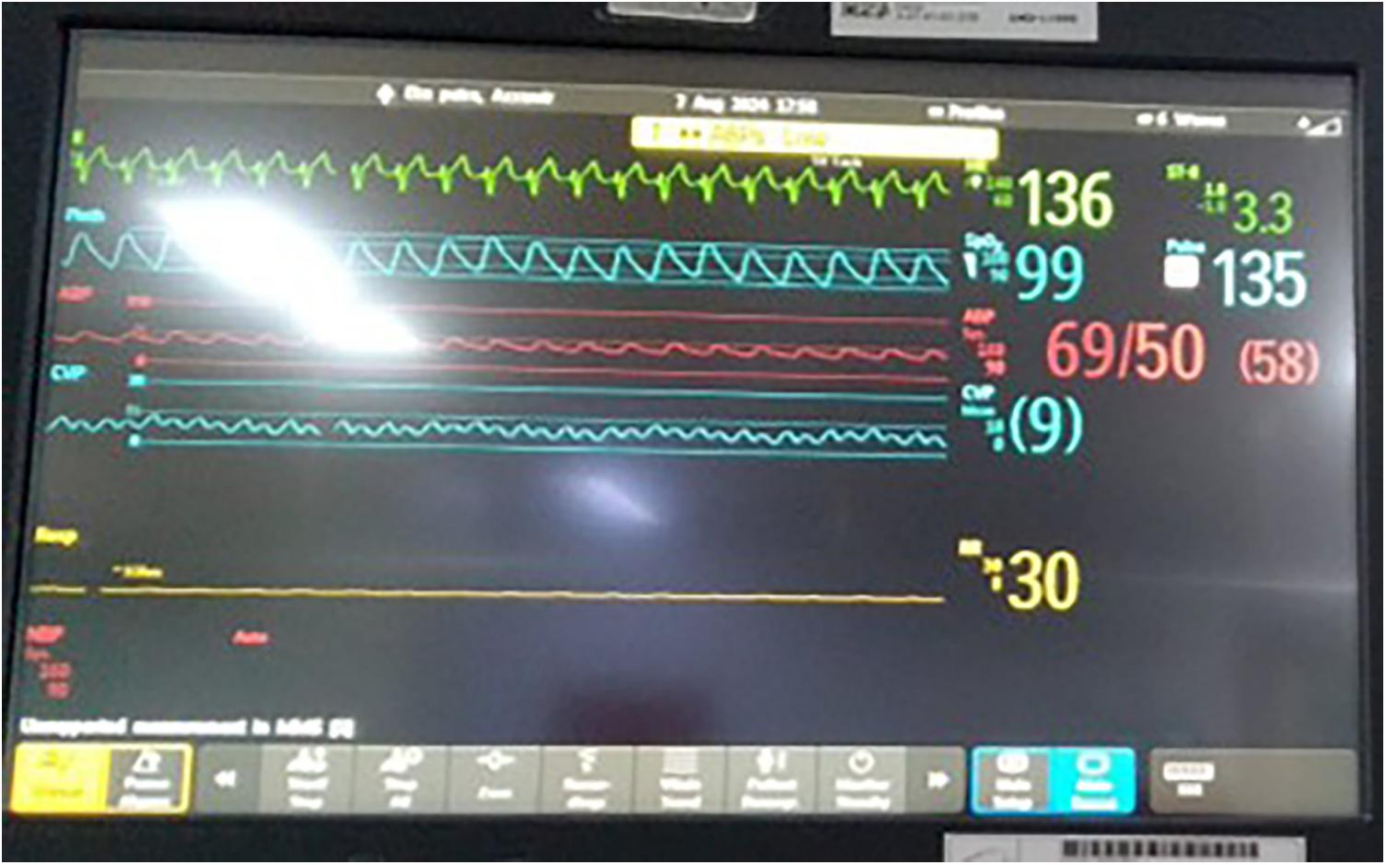
Central venous pressure (CVP) waveform showing cannon A waves and loss of atrioventricular synchrony, consistent with junctional ectopic tachycardia. The tracing supports the diagnosis in conjunction with surface ECG findings.

Immediate postoperative findings included hypotension, oliguria, and right ventricular dysfunction with a tricuspid annular plane systolic excursion (TAPSE) of 7.5 mm. On postoperative day 1 (POD-1), the patient developed supraventricular tachycardia refractory to adenosine and synchronized cardioversion. The rhythm pattern indicated junctional ectopic tachycardia (JET), which was managed using intravenous amiodarone, ivabradine, magnesium supplementation, core temperature adjustment, and sedation with dexmedetomidine. Electrocardiographic findings confirmed JET, characterized by narrow QRS complexes and atrioventricular dissociation (Figure 1). CVP waveform analysis revealed prominent cannon A waves and absence of coordinated atrial contraction, further supporting the diagnosis of junctional ectopic tachycardia (Figure 2).

**Figure 3:**
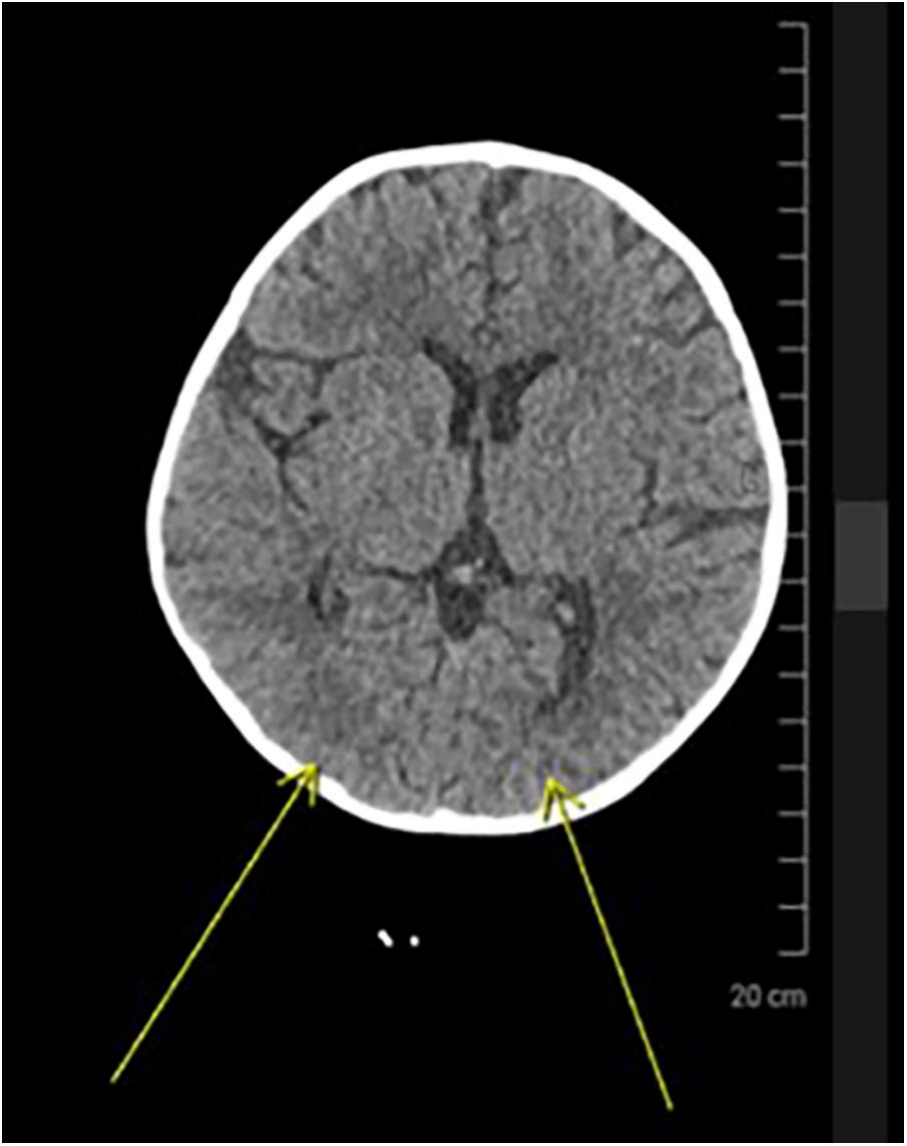
Axial brain CT showing hypodensity in the right occipital lobe (yellow arrows), consistent with embolic infarction. The revised annotation accurately indicates the infarct location.

On POD-6, echocardiography revealed a thrombus in the left atrium. Systemic anticoagulation was initiated with heparin. Brain CT performed on POD-8 revealed a hypodense lesion in the right occipital lobe, consistent with embolic infarction (Figure 3). Neuroprotective therapy included high-dose piracetam and intravenous mannitol.

By POD-17, the patient failed to establish visual contact, raising suspicion of cortical blindness. Ophthalmology and neurology consults supported the diagnosis. Partial recovery of visual tracking and spontaneous engagement was observed by POD-30, suggesting partial cortical recovery. A detailed chronological summary of postoperative events, diagnostic findings, and interventions is presented in Table 1.

**Table 1:** Clinical timeline of postoperative events.

Postoperative day (POD)	Clinical event/symptom	Diagnostic findings	Intervention/management	Outcome/notes
POD-0	Immediate post-op: hypotension, oliguria	RV dysfunction, TAPSE 7.5 mm	Fluid resuscitation, inotropes	LCOS identified
POD-1	Supraventricular tachycardia onset	ECG: JET with AV dissociation	Sedation, temperature control	Rhythm persists
POD-2	Persistent JET	—	Magnesium, ivabradine	Partial rhythm control
POD-3	Refractory JET	—	Amiodarone infusion	Gradual stabilization
POD-6	No new symptoms	Echo: left atrial thrombus	Heparin anticoagulation	Clot resolved
POD-8	Visual disengagement observed	Brain CT: right occipital infarct	Piracetam, mannitol	Cortical blindness suspected
POD-17	No visual tracking	Neurology & ophthalmology assessment	Continued neuroprotection	Cortical blindness confirmed
POD-30	Partial visual recovery	—	Supportive care	Visual tracking improved
POD-35	Discharge	Echo: TAPSE 13.5 mm, RV-FAC 38%	—	Sinus rhythm, mild PR/TR, improved vision

## Discussion

The clinical course observed in this pediatric case following complete Tetralogy of Fallot (ToF) repair exemplifies a multifaceted pathophysiological cascade, initiated by postoperative low cardiac output syndrome (LCOS). Far from being a localized cardiac complication, LCOS functioned as a systemic amplifier—precipitating junctional ectopic tachycardia (JET), left atrial thrombosis, embolic stroke, and transient cortical blindness. Each sequelae was interlinked, underscoring the vulnerability of perfusion-dependent organs and the critical importance of proactive, multidisciplinary management in pediatric cardiac postoperative care.

### Diagnostic assessment

LCOS developed secondary to right ventricular (RV) systolic dysfunction, evidenced by a reduced tricuspid annular plane systolic excursion (TAPSE) of 7.5 mm and moderate tricuspid regurgitation. This presentation is not uncommon post-ToF repair, as altered pressure-volume dynamics and geometric distortion of the RV outflow tract can impair myocardial contractility, compromise coronary perfusion, and destabilize forward flow (1). The resulting hemodynamic instability laid the groundwork for arrhythmogenic and thrombotic complications.

The emergence of JET on postoperative day 1 occurred within the well-described vulnerable 48–72 h window following cardiopulmonary bypass. JET is characterized by enhanced automaticity near the AV node, often triggered by surgical trauma, sympathetic activation, and electrolyte disturbances (2, 3). While surface ECG provided initial clues, the CVP waveform offered invasive confirmation of AV dissociation, reinforcing the diagnosis of JET in the absence of atrial ECG. In this case, as illustrated in Figure 1, the rhythm disturbance was consistent with JET, a postoperative arrhythmia commonly triggered by surgical trauma and autonomic imbalance.

### Therapeutic intervention

The arrhythmia was refractory to standard interventions such as adenosine and synchronized cardioversion, necessitating a multimodal approach including intravenous amiodarone, ivabradine, magnesium supplementation, sedation with dexmedetomidine, and core temperature modulation. These strategies align with current pediatric critical care protocols for rhythm stabilization, and highlight the importance of individualized, escalation-based management (2–4). While LCOS was initially identified prior to the onset of JET, the arrhythmia likely contributed to the persistence and worsening of low-output physiology.

On postoperative day 6, echocardiography revealed a thrombus in the left atrium—an infrequent yet serious event in pediatric post-cardiac surgical patients. The hypercoagulable milieu following cardiopulmonary bypass, compounded by stasis induced by LCOS and endothelial disruption, collectively facilitated thrombus formation (4). Unfractionated heparin therapy successfully resolved the clot, yet subsequent embolic infarction of the right occipital region underscores the complexity of managing thrombotic risks in this population (4, 5). The corrected imaging highlights the vulnerability of posterior circulation in embolic events, particularly in pediatric patients with atrial thrombus and low-output states (see Figure 3), prompting neuroprotective treatment using high-dose piracetam and intravenous mannitol (6, 7).

### Follow-up and outcomes

By postoperative day 17, failure of visual engagement raised concern for cortical blindness—a diagnosis later corroborated by neurology and ophthalmology assessments. Differential diagnosis for visual disengagement included cortical blindness, optic neuropathy, and sedation-related visual suppression. The absence of pupillary abnormalities, preserved ocular reflexes, and normal fundoscopic findings supported a cortical etiology. Cortical blindness in pediatric settings post-cardiac surgery is rare and often underrecognized, attributable to posterior cerebral artery involvement and reduced occipital perfusion (6). Importantly, progressive visual recovery observed by day 30 supports the potential reversibility of cortical ischemia, particularly when neuroprotective strategies are initiated promptly. However, this case highlights a gap in routine neurological monitoring protocols, which often overlook visual tracking and cortical responsiveness in favor of sensorium and motor assessments.

### Limitations and literature context

Overall, this case emphasizes spontaneous resolution of cortical blindness secondary to LCOS not merely as a postoperative complication but as a catalyst for multisystem decline. This cascade of complications suggest that early recognition and intervention across arrhytmogenic, thrombotic, and neurovascular domains are critical to prevent irreversible outcomes.

• **Hemodynamic monitoring** must extend beyond static pressures to include dynamic functional parameters such as TAPSE, stroke volume variation, and myocardial performance indices (1).
• **Continuous telemetry protocols** should be implemented with clear thresholds and escalation pathways for nodal arrhythmias, in accordance with AHA-endorsed rhythm surveillance guidelines (2–4).
• **Thromboprophylaxis strategies** should be individualized for patients with LCOS and cardiopulmonary bypass exposure, balancing bleeding risk and embolic potential (4, 5).
• **Neurologic evaluations** should incorporate cortical domains, including visual assessment and behavior-based tracking, to ensure comprehensive cerebral recovery screening (6, 7).

The observations made by Abdelghani et al. lend further support to the association between congenital heart surgeries—particularly those involving shunt placement or CPB—and pediatric thrombotic events (5). Mechanistically, turbulent flow, endothelial injury, and altered viscosity contribute to a prothrombotic state, particularly under LCOS conditions. While LCOS and JET are recognized postoperative risks, the emergence of cortical blindness and its subsequent resolution offer a unique perspective on pediatric neuroplasticity. This case illustrates the importance of integrating invasive monitoring, multidisciplinary diagnostics, and neuroprotective strategies in pediatric cardiac care.

## Conclusion

Low cardiac output syndrome following Tetralogy of Fallot (ToF) repair can trigger a cascade of systemic deterioration, including arrhythmias, thromboembolic events, and neurologic compromise. This case underscores LCOS not merely as a postoperative complication but as a dynamic driver of multisystem vulnerability. A timely diagnosis, proactive multidisciplinary support, and anticipatory surveillance—spanning hemodynamic, thrombotic, and neurologic domains—are essential to optimize outcomes in pediatric cardiac surgical patients. This case also highlights the potential for neurologic recovery in pediatric patients, even after embolic cortical injury.

## Data Availability

The original contributions presented in the study are included in the article/Supplementary Material, further inquiries can be directed to the corresponding author.
